# Solitaire™ with the Intention for Thrombectomy as Primary Endovascular Treatment for Acute Ischemic Stroke (SWIFT PRIME) trial: protocol for a randomized, controlled, multicenter study comparing the Solitaire revascularization device with IV tPA with IV tPA alone in acute ischemic stroke

**DOI:** 10.1111/ijs.12459

**Published:** 2015-03-16

**Authors:** Jeffrey L Saver, Mayank Goyal, Alain Bonafe, Hans-Christoph Diener, Elad I Levy, Vitor M Pereira, Gregory W Albers, Christophe Cognard, David J Cohen, Werner Hacke, Olav Jansen, Tudor G Jovin, Heinrich P Mattle, Raul G Nogueira, Adnan H Siddiqui, Dileep R Yavagal, Thomas G Devlin, Demetrius K Lopes, Vivek Reddy, Richard du Mesnil de Rochemont, Reza Jahan

**Affiliations:** 1 Department of Neurology and Comprehensive Stroke Center, University of California Los AngelesLos Angeles, CA, USA; 2 Department of Radiology, University of CalgaryCalgary, AB, Canada; 3 Department of Clinical Neurosciences, University of CalgaryCalgary, AB, Canada; 4 Department of Neuroradiology, Hôpital Gui-de-ChauliacMontpellier, France; 5 Department of Neurology, University Hospital of University Duisburg-EssenEssen, Germany; 6 Department of Neurosurgery, State University of New York at BuffaloBuffalo, NY, USA; 7 Division of Neuroradiology, Toronto Western HospitalToronto, ON, Canada; 8 Department of Neurology and Neurological Sciences, Stanford University School of MedicineStanford, CA, USA; 9 Department of Diagnostic and Therapeutic Neuroradiology, University Hospital of ToulouseToulouse, France; 10 Saint Luke's Mid America Heart Institute and University of Missouri-Kansas City School of MedicineKansas City, MO, USA; 11 Department of Neurology, University of HeidelbergHeidelberg, Germany; 12 Department of Radiology and Neuroradiology, Christian-Albrechts-University KielKiel, Germany; 13 Department of Neurology, University of Pittsburgh Medical CenterPittsburgh, PA, USA; 14 Department of Neurology, Inselspital, University of BernBern, Switzerland; 15 Marcus Stroke and Neuroscience Center, Grady Memorial Hospital, Department of Neurology, Emory University School of MedicineAtlanta, GA, USA; 16 Department of Neurosurgery, Toshiba Stroke and Vascular Research Center, University at Buffalo State University of New York at BuffaloBuffalo, NY, USA; 17 Department of Neurology and Neurosurgery, University of Miami Miller School of Medicine/Jackson Memorial HospitalMiami, FL, USA; 18 Division of Neurology, Erlanger Hospital at University of TennesseeChattanooga, TN, USA; 19 Department of Neurosurgery, Rush University Medical CenterChicago, IL, USA; 20 Institute of Neuroradiology, Klinikum der Goethe-UniversitätFrankfurt, Germany; 21 Division of Interventional Neuroradiology, University of California Los AngelesLos Angeles, CA, USA

**Keywords:** acute ischemic stroke, clinical trial, endovascular, recanalization, stent retriever, thrombolysis

## Abstract

**Rationale:**

Early reperfusion in patients experiencing acute ischemic stroke is critical, especially for patients with large vessel occlusion who have poor prognosis without revascularization. Solitaire™ stent retriever devices have been shown to immediately restore vascular perfusion safely, rapidly, and effectively in acute ischemic stroke patients with large vessel occlusions.

**Aim:**

The aim of the study was to demonstrate that, among patients with large vessel, anterior circulation occlusion who have received intravenous tissue plasminogen activator, treatment with Solitaire revascularization devices reduces degree of disability 3 months post stroke.

**Design:**

The study is a global multicenter, two-arm, prospective, randomized, open, blinded end-point trial comparing functional outcomes in acute ischemic stroke patients who are treated with either intravenous tissue plasminogen activator alone or intravenous tissue plasminogen activator in combination with the Solitaire device. Up to 833 patients will be enrolled.

**Procedures:**

Patients who have received intravenous tissue plasminogen activator are randomized to either continue with intravenous tissue plasminogen activator alone or additionally proceed to neurothrombectomy using the Solitaire device within six-hours of symptom onset.

**Study Outcomes:**

The primary end-point is 90-day global disability, assessed with the modified Rankin Scale (mRS). Secondary outcomes include mortality at 90 days, functional independence (mRS ≤ 2) at 90 days, change in National Institutes of Health Stroke Scale at 27 h, reperfusion at 27 h, and thrombolysis in cerebral infarction 2b/3 flow at the end of the procedure.

**Analysis:**

Statistical analysis will be conducted using simultaneous success criteria on the overall distribution of modified Rankin Scale (Rankin shift) and proportions of subjects achieving functional independence (mRS 0–2).

## Introduction

Stroke is the second leading cause of death and a leading cause of disability worldwide (1). Cerebral infarction, due to thrombotic occlusion of a brain artery, is the most common stroke type, accounting for 65–85% of all cases. The only specific therapy of demonstrated benefit for acute ischemic stroke (AIS) is intravenous (IV) fibrinolysis with tissue plasminogen activator (tPA) up to 4·5 hours after onset. However, patients with occlusions of large, proximal, intracranial arteries are often not responsive to IV tPA, as lytic therapy achieves early reperfusion in only 13–50% of patients with occlusions in the carotid terminus and the M1 segment of the middle cerebral artery (MCA) (2–5).

The Solitaire™ Flow Restoration (FR) device is a self-expanding stent retriever that restores blood flow in patients experiencing ischemic stroke because of large intracranial vessel occlusion. In multicenter registries and large clinical series, the Solitaire stent retriever has yielded high rates of reperfusion and favorable clinical outcomes (6–8). In a randomized, head-to-head device trial, compared with first-generation, coil retriever devices, use of the Solitaire™ FR was associated with superior recanalization rates, faster achievement of reperfusion, reduced intracranial haemorrhage complications, and improved final disability outcome (9).

The Solitaire™ with the Intention for Thrombectomy as Primary Endovascular Treatment for Acute Ischemic Stroke (SWIFT PRIME) trial is being undertaken to establish the safety and efficacy of neurothrombectomy with Solitaire in conjunction with IV tPA vs. IV tPA alone, among AIS patients treatable within six-hours of symptom onset.

## Methods

### Objective

The aim of this study is to determine whether subjects experiencing an AIS due to large vessel occlusion treated with combined IV tPA and Solitaire revascularization device within six-hours of symptom onset have less stroke-related disability than those subjects treated with IV tPA alone.

### Design

**Figure 1:**
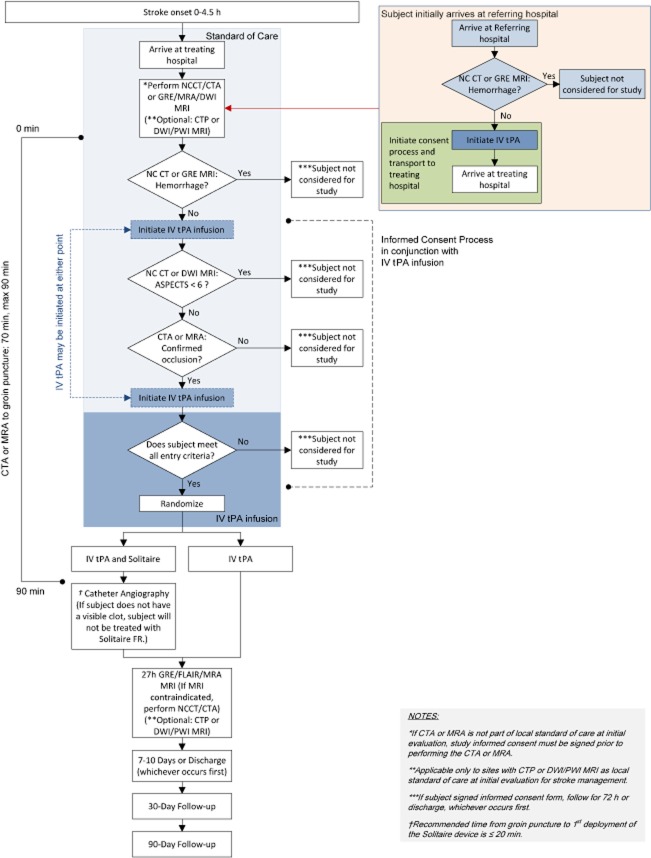
Study design diagram. ASPECTS, Alberta Stroke Program Early CT score; CTA, computed tomography angiography; CTP, computed tomography perfusion imaging; DWI, diffusion-weighted imaging; FLAIR, fluid attenuated inversion recovery; FR, Flow Restoration; GRE, gradient refocused echo; IV, intravenous; MRA, magnetic resonance angiography; MRI, magnetic resonance imaging; NCCT, non-contrast CT; PWI, perfusion weighted imaging; tPA, tissue plasminogen activator.

The study is a global, multicenter, two-arm, prospective, randomized, open, blinded end-point clinical trial. The study patient flow outline is shown in Fig. 1.

### Patient population

Entry criteria were structured to enroll patients who have AIS, moderate to severe neurologic deficits, harbor imaging-confirmed occlusions of proximal, anterior circulation arteries, do not have a large, established core infarct, have received treatment with IV tPA, and are able to undergo Solitaire neurothrombectomy within six-hours of last known well time. Full, detailed study inclusion and exclusion criteria are shown in Table 1.

**Table 1:** Study inclusion and exclusion criteria

Inclusion criteria:
1. Age 18 – 80
2. Clinical signs consistent with acute ischemic stroke
3. Prestroke Modified Rankin Score ≤ 1
4. NIHSS ≥ 8 and < 30 at the time of randomization
5. Initiation of IV tPA within 4·5 hours of onset of stroke symptoms (onset time is defined as the last time when the patient was witnessed to be at baseline), with investigator verification that the subject has received/is receiving the correct IV tPA dose for the estimated weight prior to randomization.
6. Thrombolysis in cerebral infarction (TICI) 0–1 flow in the intracranial internal carotid, M1 segment of the MCA, or carotid terminus confirmed by CT or MR angiography that is accessible to the Solitaire™ FR device. (Note: M1 segment of the MCA is defined as the arterial trunk from its origin at the ICA to the first bifurcation or trifurcation into major branches neglecting the small temporo-polar branch.)
7. Subject is able to be treated within six-hours of onset of stroke symptoms and within 1·5 hours (90 min) from CTA or MRA to groin puncture.
8. Subject is willing to conduct protocol-required follow-up visits.
9. An appropriate signed and dated informed consent form (or enrollment under exception from explicit informed consent if permitted under country regulations)
10. Subject is affiliated with a social security system (if required by individual country regulations).
11. Subject meets national regulatory criteria for clinical trial participation.
Exclusion criteria:
1. Subject who is contraindicated to IV tPA as per local national guidelines.
2. Female who is pregnant or lactating or has a positive pregnancy test at time of admission.
3. As applicable by French law, subject who is a protected individual such as an incompetent adult or incarcerated person.
4. Rapid neurological improvement prior to study randomization suggesting resolution of signs/symptoms of stroke
5. Known serious sensitivity to radiographic contrast agents
6. Known sensitivity to nickel, titanium metals, or their alloys
7. Current participation in another investigation drug or device treatment study
8. Known hereditary or acquired haemorrhagic diathesis, coagulation factor deficiency. (A subject without history or suspicion of coagulopathy does not require INR or prothrombin time lab results to be available prior to enrollment.)
9. Renal failure as defined by a serum creatinine > 2·0 mg/dl (or 176·8 μmol/l) or glomerular filtration rate (GFR) < 30.
10. Subject who requires hemodialysis or peritoneal dialysis, or who has a contraindication to an angiogram for whatever reason.
11. Life expectancy of less than 90 days
12. Clinical presentation suggests a subarachnoid haemorrhage, even if initial CT or MRI scan is normal
13. Suspicion of aortic dissection
14. Subject with a comorbid disease or condition that would confound the neurological and functional evaluations or compromise survival or ability to complete follow-up assessments.
15. Subject currently uses or has a recent history of illicit drug(s) or abuses alcohol (defined as regular or daily consumption of more than four alcoholic drinks per day).
16. Known history of arterial tortuosity, preexisting stent, and/or other arterial disease that would prevent the device from reaching the target vessel and/or preclude safe recovery of the device
Imaging exclusion criteria
1. CT or MRI evidence of haemorrhage on presentation
2. CT or MRI evidence of mass effect or intra-cranial tumour (except small meningioma)
3. CT or MRI evidence of cerebral vasculitis
4. CT showing hypodensity or MRI showing hyperintensity involving greater than 1/3 of the MCA territory (or in other territories, > 100 cc of tissue) on presentation
5. *Baseline non-contrast CT or DWI MRI evidence of a moderate/large core defined as extensive early ischemic changes of Alberta Stroke Program Early CT score (ASPECTS) < 6.
6. CT or MRI evidence of a basilar artery (BA) occlusion or posterior cerebral artery (PCA) occlusion
7. CTA or MRA evidence of carotid dissection or complete cervical carotid occlusion requiring stenting at the time of the index procedure (i.e. mechanical thrombectomy)
8. Imaging evidence that suggests, in the opinion of the investigator, the subject is not appropriate for mechanical thrombectomy intervention (e.g. inability to navigate to target lesion, moderate/large infarct with poor collateral circulation, etc.).

* Before Revision F, this criterion stated: ‘Core Infarct and hypoperfusion: a) MRI- or CT-assessed core infarct lesion greater than 50 cc; b) Severe hypoperfusion lesion (10 sec or more Tmax lesion larger than 100 cc); c) Ischemic penumbra < 15 cc and mismatch ratio ≤1·8’.

CT, computed tomography; CTA, computed tomography angiography; DWI, diffusion-weighted imaging; ICA, internal carotid artery; INR, international normalized ratio; IV, intravenous; MCA, middle cerebral artery; MRA, magnetic resonance angiography; MRI, magnetic resonance imaging; NIHSS, National Institutes of Health Stroke Scale; tPA, tissue plasminogen activator.

During the course of the trial, a revision in imaging entry criteria was made. At launch, the study employed a ‘target mismatch’ strategy, using multimodal computed tomography (CT) or magnetic resonance imaging (MRI), including perfusion sequences, to identify patients with salvageable tissue (10). Subsequently, to accommodate sites with limited perfusion imaging capability and ensure accelerated treatment delivery (11), Revision F of the study changed imaging entry criteria to employ a ‘small to moderate core’ strategy, using Alberta Stroke Program Early CT score (ASPECTS) ratings of CT or magnetic resonance (MR) images (12).

At sites in which standard imaging practice includes CT or MR angiography (MRA) (and, under the initial study entry criteria, perfusion imaging), patients may directly be enrolled in the randomized phase of the trial. At sites where this imaging is not standard practice, subjects are first enrolled in a screening phase and the imaging studies are obtained. A subject is considered enrolled into the screening phase of the study after the informed consent form has been signed or country-specific requirements have been met for enrollment without explicit informed consent in emergency circumstances. A subject is considered enrolled in the randomized phase of the study after a treatment allocation is assigned from the randomization system.

### Randomization

Subjects will be randomly assigned in a 1 : 1 fashion to one of two treatment arms: (1) IV tPA and Solitaire device or (2) IV tPA alone. The number of treatments and controls will be balanced within investigational sites and by baseline National Institutes of Health Stroke Scale (NIHSS) severity (≤17 vs. >17), age (<70 years vs. ≥70 years at the time of randomization), and occlusion location (M1 vs. all other). Subject allocation to treatment will be accomplished by using an interactive web response or interactive voice response system.

### Intervention

#### Endovascular treatment

**Figure 2:**
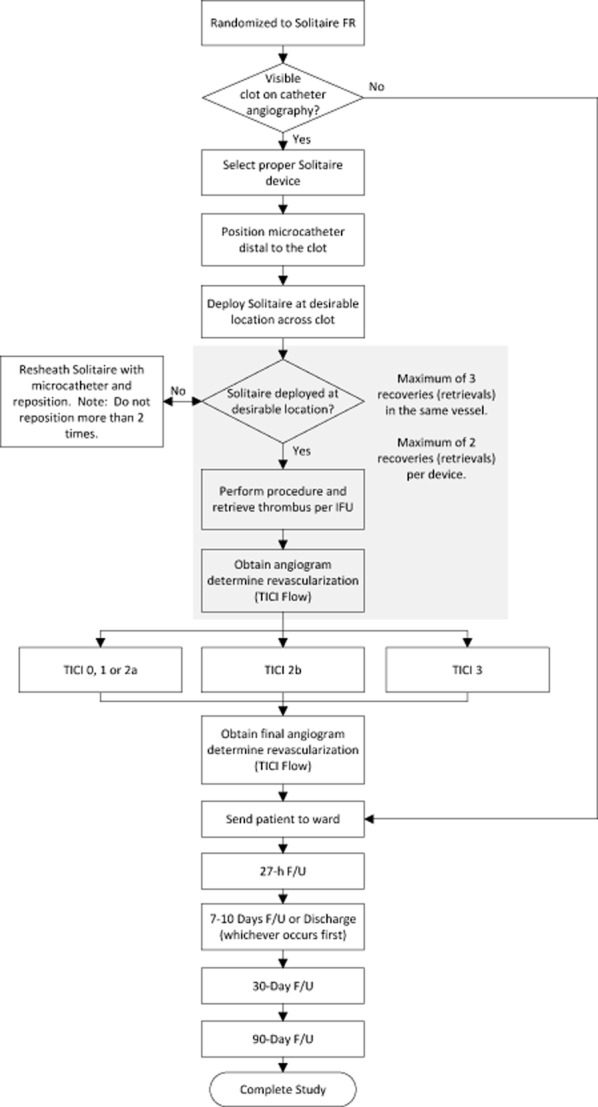
Solitaire revascularization device procedure. FR, Flow Restoration; FU, follow-up; IFU, instructions for use; TICI, thrombolysis in cerebral infarction.

The Solitaire device revascularization procedure is summarized in Fig. 2. Among endovascular arm patients, to facilitate speed of intervention, two target procedural time targets are employed. Time from acquisition of the final study-qualifying image (perfusion CT or MR sequence prior to Revision F; CT or MRA sequence under Revision F) to groin puncture is optimally targeted to be less than 70 min and should be no greater than 90 min. Time from groin puncture to first deployment of the Solitaire device is targeted to be less than 20 min.

For the intervention, the proceduralist may use the Solitaire™ FR device or the Solitaire™ 2 revascularization device. The proper study device size is selected per device-specific instructions for use. If deemed appropriate by the neurointerventionalist, IV sedation or general anesthesia may be administered to assure subject comfort and safety. Several retrieval passes with Solitaire revascularization devices, if needed, may be performed, up to: (1) a maximum of three retrievals in the same vessel and (2) a maximum of two retrievals per device.

#### Procedural angiography

Among subjects randomized to IV tPA + Solitaire device group, angiography images obtained during the procedure will consist of the following: (1) *Baseline angiogram:* obtained prior to device deployment while assessing the clot location; (2) *Post-device use angiogram:* obtained immediately after each pass of study device use; and (3) *Final post-procedural angiogram:* obtained after all treatments have been completed.

#### CT or MRI

At screening, non-contrast CT (NCCT) or gradient refocused echo MRI are used to exclude haemorrhage, NCCT or diffusion-weighted imaging (DWI) MRI to identify early ischemic changes using the ASPECTS scale, and CT angiography (CTA) or MRA to assess for target vascular occlusion. In patients transferred from another facility, updated screening imaging at the study hospital must be obtained to qualify the patient for the trial.

At all sites prior to Revision F and with Revision F at sites where perfusion CT or MR imaging is local standard of care, perfusion images will also be obtained at screening and will be processed locally using RApid processing of PerfusIon and Diffusion (RAPID), an operator-independent system for processing of perfusion weighted imaging (PWI) and DWI images (13). RAPID will generate PWI and DWI maps, segment the PWI and DWI lesions, and calculate lesion volumes within 10 min of scan completion.

Follow-up multimodal CT or MRI will be obtained among all study patients at 27 h (±6 h) from time of randomization to assess any presence of haemorrhage, recanalization of the occluded artery, reperfusion of the ischemic region, and infarct growth. At all sites prior to Revision F and with Revision F at sites where perfusion CT or MR imaging is local standard of care, perfusion images will also be acquired at 27 h.

#### Follow-up and assessment of clinical outcomes

Table 2 shows the schedule of study visits. A 27-h post-randomization visit includes an NIHSS examination and CT or MR imaging. Subsequent follow-up visits occur at 7–10 days (or discharge if earlier), 30 days, and 90 days, and include the modified Rankin Scale (mRS) assessing global disability, the Barthel Index assessing instrumental activities of daily living, the NIHSS exam assessing neurologic deficit, and the European Quality of Life Scale (EuroQoL) assessing health-related quality of life. In addition, to assess health economics outcomes, a Resource Utilization Questionnaire will collect information on use of healthcare resources through 90 days, including the index hospitalization, subsequent inpatient and outpatient care, and readmissions.

**Table 2:** Study visits

Assessment method	Screening	Procedure	27 h post-randomization	7–10 days or discharge	30-day follow-up	90-day follow-up	Unscheduled follow-up
Range	0	0	± 6 h	0	± 7 days	± 15 days	N/A
Pregnancy test	w	–	–	–	–	–	–
Blood labs	w	–	–	–	–	–	–
NIH Stroke Scale	w	–	w	w	w	w	w
Prestroke modified Rankin Scale	w	–	–	–	–	–	–
Modified Rankin Scale	–	–	–	w	w	w	w
Barthel Index	–	–	–	w	–	w	w
MRI or NCCT	w	–	w	–	–	–	–
ASPECTS	w	–	–	–	–	–	–
MRA or CTA	w	–	w	–	–	–	–
Catheter angiography	–	w	–	–	–	–	–
Resource utilization	–	–	–	w	w	w	w
EuroQol 5D-5L	–	–	–	–	w	w	–
Concomitant medications	w	w	w	w	w	w	w
Adverse events	w	ww	w	w	w	w	w
Optional: Applicable only to sites with DWI/PWI or CTP imaging as local standard of care at initial evaluation:							
PWI MR or CT perfusion	w	–	w	–	–	–	–
RAPID imaging processing	w	–	w	–	–	–	–

ASPECTS, Alberta Stroke Program Early CT score; CT, computed tomography; CTA, computed tomography angiography; EuroQoL, European Quality of Life Scale; MR, magnetic resonance; MRA, magnetic resonance angiography; MRI, magnetic resonance imaging; NCCT, non-contrast computed tomography; NIH, National Institutes of Health; RAPID, RApid processing of PerfusIon and Diffusion.

#### Adverse event categorization

All adverse events will be validated and categorized for severity and relatedness by a clinical events committee, comprised of three expert physicians independent of the investigational sites. Relatedness categories will include: (1) study disease-related: event clearly attributable to underlying disease state with no temporal relationship to the device, treatment, or medication; (2) concomitant disease-related: event attributable to disease other than the study disease with no temporal relationship to the device, treatment, or medication; (3) IV tPA-related: event clearly attributable to IV tPA medication with no temporal relationship to the device or treatment; (4) procedure-related: event has strong temporal relationship to the procedure or treatment of the device implantation or any user handling; (5) primary study device-related: event has a strong temporal relationship to the Solitaire device and alternative etiology is less likely; (6) ancillary device-related: any device other than Solitaire™ FR, such as microcatheter or guidewire; (7) device unknown: device related but unable to attribute a specific device; (8) other; and (9) unknown.

#### Imaging core laboratory

The Core Imaging Laboratory will provide an independent and unbiased assessment of imaging-related entry criteria and end-points. Entry criteria assessed by the Core Lab on initial CT or MRI will include presence of small core using ASPECTS score, presence of target penumbral pattern, and presence of proximal vessel occlusion in the internal carotid artery (ICA) or M1 MCA. Among endovascular arm patients, the Core Lab will assess the catheter angiograms for vascular reperfusion following device use, measured by the thrombolysis in cerebral infarction (TICI) scale. On 27-h CT or MRI scans, the Core Lab will assess outcome ASPECTS score, infarct volume, recanalization defined by CTA or MRA TICI grades, and presence of haemorrhagic transformation. In patients undergoing perfusion imaging at 27 h, the Core Lab will assess infarct volume and reperfusion ratio.

Haemorrhages on CT/MRI will be radiologically classified according to the following categories: HT 1 – small petechiae within ischemic field without mass effect; HT 2 – confluent petechiae within ischemic field without mass effect; PH1 – hematoma within ischemic field with some mild space-occupying effect but involving ≤ 30% of the infarcted area; PH2 – hematoma within ischemic field with space-occupying effect involving > 30% of the infarcted area; RIH – any intraparenchymal haemorrhage remote from the ischemic field; IVH – intraventricular haemorrhage; and SAH – subarachnoid haemorrhage.

All Core Lab readings will be performed independently by two experienced readers. For key measures, discrepancies will be resolved by a third, independent reader. The Core Lab will assess all CT and MR imaging blinded to treatment assignment. Assessment of catheter angiographic images for revascularization will not be blinded, as this evaluation will only be done for subjects in the IV tPA plus Solitaire FR treatment arm.

#### Primary outcome

The primary study end-point is the degree of disability or dependence at 90 days as assessed by the mRS. A global measure of disability, the mRS comprises of seven grades ranging from 0 (no symptoms) to 6 (death). The mRS will be assessed in a formally operationalized manner by use of the Rankin Focused Assessment – Ambulation (RFA-A). The 90-day mRS will be assessed by study personnel certified in the scoring of the mRS using the RFA-A and will be blinded to treatment assignment.

#### Secondary outcomes

The study has three secondary clinical efficacy end-points: (1) death due to any cause at 90 days; (2) functional independence as defined by mRS score ≤ 2 at 90 days; and (3) change in NIHSS score at 27 ± 6 h post-randomization.

The study has four technical efficacy end-points: (1) volume of cerebral infarction as measured by a CT or MRI scan at 27 ± 6 h post-randomization; (2) reperfusion measured by reperfusion ratio on CT or MRI scan 27 ± 6 h post-randomization; (3) arterial revascularization measured by TICI 2b or 3 following device use; and (4) correlation of RAPID-assessed core infarct volume with 27 ± 6 h post-randomization stroke infarction in subjects who achieved TICI 2b–3 reperfusion without intracranial haemorrhage.

Study safety end-points are: (1) all serious adverse events and (2) symptomatic intracranial haemorrhage (SICH) at 27 ± 6 h post-randomization. SICH is defined as any PH1, PH2, RIH, SAH, or IVH associated with a four-point or more worsening on the NIHSS within 24 h.

Health economic evaluation end-points will include features of care for the index stroke (length of stay, discharge to home or any other type of facility and cost of that care, rehabilitation services, home health services), readmissions due to subsequent stroke, and calculation of quality-adjusted life year assessment, using utilities derived from the EuroQoL 5D-5L assessment. In conjunction with external data on long-term stroke outcomes, these data will be used to estimate the incremental cost-effectiveness of Solitaire treatment for the target population.

#### Sample size

The primary effectiveness end-point in this study is 90-day global disability assessed via the blinded evaluation of mRS, analyzed using simultaneous success criteria on: (1) the overall distribution of mRS (Rankin shift) and (2) the proportion of subjects achieving functional independence, defined as mRS of 0 to 2.

The statistical hypothesis on Rankin shift is that the distribution of mRS in subjects randomized to the IV tPA plus Solitaire will be more favorable than the distribution in the IV tPA only group. For this purpose, the entire distribution 0 to 6 of mRS values will be considered except that categories 5 and 6 are collapsed into a single group. Additionally, to measure benefit in terms of functional independence, a simultaneous requirement for success is that the difference in the proportion of patients with mRS 0–2 outcomes nominally meets a prespecified minimum dependent on the evaluable sample size at the time of the assessment.

Power and sample size are determined by the mRS shift analysis and are computed by assuming that the true proportions of subjects with various mRS outcomes at the 90-day follow-up visit are as presented in Table 3. The tPA-only outcome distributions are those observed with IV tPA use in the two NINDS (National Institute of Neurologic Disorders and Stroke) tPA trials, restricted to those subjects with baseline NIHSS of at least 8 but less than 30 to correspond with the current study's inclusion criteria. The Solitaire outcome distributions are based on data collected in heterogeneous settings including the use and non-use of IV tPA. Included in these rates is adjustment for an expected proportion of 10% of subjects randomized to the Solitaire group who will be unable for anatomical reasons to be treated with the randomized device; this cohort of subjects will remain in the Solitaire group per intent to treat but are assumed to have outcomes similar to those randomized to tPA only.

**Table 3:** Hypothesized true outcomes for sample size calculations

Randomized group	mRS 0	mRS 1	mRS 2	mRS 3	mRS 4	mRS 5–6
Solitaire plus IV tPA	19·7%	18·2%	18·3%	9·4%	12·5%	21·9%
IV tPA alone	11·0%	21·6%	8·1%	15·7%	14·4%	29·2%

IV, intravenous; mRS, modified Rankin Scale; tPA, tissue plasminogen activator.

Sample size and power are computed incorporating a group sequential analysis plan with five interim analyses for efficacy, futility, and safety. Under this group sequential analysis plan, with a one-sided alpha level set at 0·025, 750 subjects with an evaluable primary endpoint provide 90% power for testing the study's primary effectiveness hypothesis; assuming attrition of 10% for the primary end-point, the total randomized sample size is up to 833 while the expected randomized sample size under the alternative hypothesis is approximately 477 (Table 4).

**Table 4:** Group sequential analysis

Evaluable sample size	Stopping for safety	Stopping for efficacy		Stopping for futility	
	Two-sided alpha for mortality	Two-sided alpha for Rankin shift	Effect size Δ for mRS 0–2	Effect size φ for mRS mean value	Effect size Δ for mRS 0–2
200	0·0036	0·0200	12·0%	0·00	0·0%
300	0·0058	0·0125	10·0%	0·00	0·0%
400	0·0094	0·0150	9·0%	0·10	n/a
500	0·0147	0·0150	8·0%	0·14	n/a
600	0·0203	0·0150	6·0%	0·14	n/a
750 (final)	0·0340	0·0350	5·0%	n/a	n/a

mRS, modified Rankin Scale.

### Statistical analyses

Statistical testing of the primary end-point will be conducted using the Cochran–Mantel–Haenszel test for the shift in Rankin scores, augmented by the requirement of functional independence cited above. Type I and Type II error will be computed via simulation and overall alpha will be controlled at a one-sided level of 0·025.

The primary end-point analysis and the testing strategy based on the group sequential design will be conducted using the intention to treat (ITT) population. If the results are favorable, a second analysis of the primary end-point will be conducted using the Food and Drug Administration (FDA) evaluable cohort (those having received IV tPA within three-hours of stroke symptom onset) following a step-down approach.

Group Sequential Analysis: Interim analyses will be performed when 200 subjects from the ITT population have provided evaluable primary effectiveness data and then after each subsequent 100 subjects, to a maximum of 750 subjects with evaluable data (i.e. 200, 300, 400, 500, 600, and 750). Table 4 provides the group sequential boundaries including minimum acceptable statistical criteria at each look (including the final analysis). Additionally, a safety stopping rule is defined under which the trial is halted for a substantial mortality difference in either possible direction at the various interim looks. This rule does not impact effectiveness findings.

### Study organization and funding

Study conduct is overseen by the executive and steering committees and the sponsor. The executive committee will be led by the global principal investigator (PI) and will include a global imaging and workflow PI, an EU (European Union) national PI, US and EU interventional PIs, and an interventional advisor. The steering committee will be comprised of recognized experts in the treatment of stroke and representatives from leading enrolling sites. The executive committee, assisted by the steering committee, will oversee all clinical trial activities including protocol development and protocol amendment during study conduct. Covidien is the study sponsor and source of funding, and will perform monitoring at each site to ensure protection of the rights of subjects, the safety of subjects, and the quality and integrity of the data collected and submitted according to FDA and EMA (European Medicines Agency) regulation.

### Data Safety Monitoring Board

The Data Safety Monitoring Board will be comprised of individuals who are independent of the investigational sites and who have expertise in multiple disciplines, including neurology, neurosurgery, interventional neuroradiology, and biostatistics/epidemiology. The independence of the members will be maintained and bias minimized by blinding, to the extent possible, all members to individual subject and treating center identity when reviewing study data. This board shall provide recommendations to the sponsor regarding stopping/continuing enrollment in the study, including carrying out planned formal interim analyses.

## Discussion

The SWIFT PRIME trial will provide definitive information on the efficacy and safety of the Solitaire revascularization devices when added to IV tPA, in comparison with therapy with IV tPA alone. The study was designed to incorporate lessons from trials of earlier generation endovascular interventions that failed to demonstrate treatment benefit (14–17). Unlike those prior studies, SWIFT PRIME tests a highly effective recanalization device, the Solitaire stent retriever, which achieves recanalization much more frequently and rapidly than earlier generation therapies (9). SWIFT PRIME ensures that all enrolled patients have appropriate target occlusions by mandating CTA or MRA imaging at entry. SWIFT PRIME is enrolling only patients with occlusion locations that are distinctively responsive to endovascular therapy, by including ICA and M1 MCA lesions that respond poorly to IV tPA and excluding M2 and more distal MCA lesions that frequently benefit from IV tPA alone. SWIFT PRIME is identifying patients who have salvageable brain left to save at the time of enrollment by requiring small core size on ASPECTS or RAPID imaging. SWIFT PRIME is ensuring that all patients who clinicians expect may respond to therapy will be enrolled by requiring that sites commit to enroll all eligible study patients. SWIFT PRIME is minimizing progression of infarct that occurs during delays between the time of study enrollment and endovascular reperfusion through rigorous focus on management efficiency and intensive quality improvement, training, and feedback about interventional workflow during the trial. Lastly, SWIFT PRIME is providing equivalent concomitant therapy in the two treatment arms, using full-dose tPA in both study groups, rather than a reduced dose in the neuroendovascular arm.

SWIFT PRIME enrolled its first patient on December 30, 2012. When completed, SWIFT PRIME will provide pivotal data allowing assessment of the efficacy and safety of the Solitaire revascularization device for reperfusion of AIS caused by large intracranial vessel occlusion.

## Study personnel

### Global PI

Jeffrey L. Saver, MD

### Executive committee

Hans-Christoph Diener, MD (EU PI), Mayank Goyal, MD (global imaging and workflow PI), Elad Levy, MD (US interventional PI), Alain Bonafé, MD (EU interventional PI), Vitor Mendes Pereira, MD (EU interventional PI), and Reza Jahan, MD (interventional advisor)

### Steering committee

Gregory W. Albers, MD (global penumbral imaging investigator), Christophe Cognard, MD, David Cohen, MD, Werner Hacke, MD, Olav Jansen, MD, Tudor G. Jovin, MD, Heinrich P. Mattle, MD, Raul G. Nogueira, MD, Adnan H. Siddiqui, MD, and DiLeep R. Yavagal, MD

### Data safety and monitoring committee

Rüdiger von Kummer, MD (chair), Wade Smith, MD, Francis Turjman, MD, and Scott Hamilton, PhD

### Clinical events committee

Arun Amar, MD (chair), Nerses Sanossian, MD, and Yince Loh, MD

### Site investigators and coordinators

S. Starkman, MD (PI), J. Guzy (UCLA/Ronald Reagan UCLA Medical Center); W. Clark, MD (PI), S. Jamieson (Oregon Health and Science University); V. Reddy, MD (PI), L. Baxendell (University of Pittsburgh Medical Center); A. Siddiqui, MD (PI), S. Hall (University of Buffalo Neurosurgery/Buffalo General Hospital); D. Yavagal, MD (PI), K. Ramdas (University of Miami/Jackson Memorial Hospital); T. Devlin, MD (PI), K. Barton (Chattanooga Center for Neurologic Research/Erlanger Hospital); B. Jagadeesan, MD (PI), D. Hildebrandt (Hennepin Country Medical Center); B-F. Fitzsimmons, MD (PI), T. Larson (Medical College of Wisconsin/Froedtert Memorial Lutheran Hospital); R. Ecker, MD (PI), L. Connolly (Maine Medical Center); R. Budzik, MD (PI), M. Taylor (Ohio Health Research Institute/Riverside Methodist Hospital); I. Acosta, MD (PI), E. Bonwit (Florida Hospital); E. Deshaies, MD (PI), M. Villwock (State University of New York Upstate); M. Jumaa, MD (PI), T. Hendrickson (Promedica Toledo Hospital); C. Ramsey, MD (PI), S. Renfrow (Central Baptist Hospital); M.S. Hussain, MD (PI), A. Richmond (Cleveland Clinic Cerebrovascular Center, NI); J. Carpenter, MD (PI), J. Domico (West Virginia University Hospital); V. Deshmukh, MD (PI), M. Rodriguez (Providence Brain and Spine Institute); A. Puri, MD (PI), M. Howk (University of Massachusetts Medical Center); R. Nogueira, MD (PI), S. Doppelheuer (Emory University/Grady Medical Center); D. Lopes, MD (PI), C. Anton (Rush University Medical Center); C. Martin, MD (PI), B. Brion (Saint Luke's Hospital); H. Farid, MD (PI), L. Cross (St. Jude Medical Center); A. Hassan, DO (PI), L. Jones-Fullingim (Valley Baptist Medical Center-Harlingen); A. Malek, MD (PI), M. Smith, P. Beck (Tenet Health Systems); A. Bonafé, MD (PI), M. Moynier (CHU de Montpellier – Hôpital Gui de Chauliac); O. Jansen, MD (PI), S. Krieter (Universitätsklinikum Kiel- Abteilung für Neuroradiologie); JF. Arenillas, MD (PI), FJ Reyes Muñoz (Hospital Clinico Universitario de Valladolid); R. du Mesnil de Rochemont, MD (PI), H. Braun (Klinikum der Johann Wolfgang Goethe-Universität); L. Remonda, MD (PI), M. Guglielmetti (Kantonsspital Aarau); C. Weimar, MD (PI), M. Dietzold (Universitätsklinikum Essen); K. Hansen, MD (PI), T. Hyldal (Rigshospitalet, Copenhagen University Hospital); P. Papanagiotou, MD (PI), H. Merdivan (Klinikum Bremen-Mitte); M. Killer-Oberpfalzer, MD (PI), A. Jedlitschka (Universitätsklinikum Christian Doppler Klinik Salzburg); P. Ringleb, MD (PI), I. Ludwig (Universitätsklinikum Heidelberg/ Neurologische Klinik und Poliklinik); G. Reimann, MD (PI), K. Burg (Klinikum Dortmund); C. Brekenfeld, MD (PI), G. Wortmann (Universitätsklinikum Hamburg-Eppendorf); S. Prothmann, MD (PI), B. Schwaiger (Klinikum rechts der Isar – Technische Universität München); H-P Haring, MD (PI), K. SØrensen (Landes-Nervenklinik Wagner-Jauregg); and G. Andersen, MD (PI), E. Bach (Aarhus University Hospital)
